# Gold Nanoparticle-Based Microfluidic Chips for Capture and Detection of Circulating Tumor Cells

**DOI:** 10.3390/bios13070706

**Published:** 2023-07-04

**Authors:** Valber A. Pedrosa, Kangfu Chen, Thomas J. George, Z. Hugh Fan

**Affiliations:** 1 Institute of Bioscience of Botucatu, Sao Paulo State University—Unesp, Botucatu 18603-560, Brazil; 2 Interdisciplinary Microsystems Group, Department of Mechanical and Aerospace Engineering, University of Florida, P.O. Box 116250, Gainesville, FL 32611, USA; 3 Department of Medicine, University of Florida, P.O. Box 100278, Gainesville, FL 32610, USA; tgeorge@ufl.edu; 4 J. Crayton Pruitt Family Department of Biomedical Engineering, University of Florida, P.O. Box 116131, Gainesville, FL 32611, USA

**Keywords:** circulating tumor cells, gold nanoparticles, microfluidics, cell capture

## Abstract

Liquid biopsy has progressed to its current use to diagnose and monitor cancer. Despite the recent advances in investigating cancer detection and diagnosis strategies, there is still a room for improvements in capturing CTCs. We developed an efficient CTC detection system by integrating gold nanoparticles with a microfluidic platform, which can achieve CTC capture within 120 min. Here, we report our development of a simple and effective way to isolate CTCs using antibodies attached on gold nanoparticles to the surface of a lateral filter array (LFA) microdevice. Our method was optimized using three pancreatic tumor cell lines, enabling the capture with high efficiency (90% ± 3.2%). The platform was further demonstrated for isolating CTCs from patients with metastatic pancreatic cancer. Our method and platform enables the production of functionalized, patterned surfaces that interact with tumor cells, enhancing the selective capture of CTCs for biological assays.

## 1. Introduction

Metastasis is the leading cause of death from cancer and the second leading cause of death worldwide, accounting for 8.8 million deaths [1]. Metastasis stems from primary tumors, which invade adjacent tissue, undergoing angiogenesis to form new blood vessels to nourish and maintain the cell’s activity [2]. Once there is an invasion of neighboring tissues, intravasation into the circulation may occur, reaching the interior of blood or lymphatic vessels, which disseminates throughout the body and allows cells to survive the physical stress of the environment [3,4]. Currently, detecting circulating tumor cells (CTCs) in blood has a potential to become a technology that can screen cancer noninvasively, diagnose cancer early, monitor tumor progression, and evaluate therapy responses [5,6]. The diagnosis of cancer is usually made through punctures and biopsies, where a small sample of the patient’s tumor is taken during a surgical procedure [7]. These cells are analyzed by specialists and classified according to tissue and cellular morphology by the presence of already established markers that allow the correct identification of the tumor. However, the traditional tissue removal method via biopsy requires highly trained personnel and high cost. Furthermore, it is painful to patients and is time-consuming. Its other limitations are related to (1) tumor heterogeneity and (2) the fact that it is not always feasible or repeatable; it provides information limited to a single point in space and time, thus failing to capture the complex tumor heterogeneity. However, developing noninvasive methods to detect and monitor tumors remains a significant challenge and unmet clinical need in oncology.

It is worth mentioning that detecting circulating tumor cells (CTCs) pose a significant challenge due to their rarity. Typically, only one to a few CTCs are present in 1 mL of patient’s blood, which contains about 7.8 million white blood cells and five billion red blood cells [7]. To be successful, the field requires the development of high-efficiency and high-purity methods to detect CTCs in blood samples, which can aid in faster decision making and a better understanding of the type of tumor the patient has. Alternative detecting approaches based on antibodies [8,9,10] and aptamers [11,12,13,14], as well as those based on ct-DNA [15,16], have been reported for liquid biopsy using patient blood. One CTC method and a couple of ctDNA assays have received approval from the US Food and Drug Administration (FDA) for clinical use, providing efficient and specific methods for liquid biopsy [14,15,16,17]. However, despite their effectiveness, some CTC techniques cannot study interactions between cells and the ligand-coated surface due to the large surface-to-volume ratio and diffusion distance. As such, further advancements are needed to minimize these limitations and enhance the accuracy of CTC isolation methods.

The isolation and detection of circulating tumor cells (CTCs) have been made possible through a variety of techniques, including immunomagnetic beads [18], size-based filtration systems [19], and microfluidic devices [20,21]. Of these techniques, microfluidics has emerged as a promising approach due to their ability to handle small blood volumes and provide high sensitivity and specificity. Detecting CTCs using microfluidics involves passing a blood sample through a device designed to capture and identify CTCs, which may use antibody-coated surfaces or microfluidic structures that mimic blood vessels to isolate and trap CTCs. Once captured, CTCs can be analyzed using various methods such as imaging and drug sensitivity testing, providing valuable insights into the type and progression of cancer, as well as the potential effectiveness of specific treatments [21].

Using nanoparticles in conjunction with microfluidics has greatly improved the sensitivity and specificity of CTC detection [22,23,24]. Nanoparticles offer a unique platform for biomarker detection due to their easy synthesis, surface chemistry, and biocompatibility. Here, we investigated the use of gold nanoparticles on a lateral filter array (LFA) to capture and release cancer cells from whole blood. Gold nanoparticles were functionalized with anti-EpCAM antibodies to coat a microfluidic device, allowing for the specific capture of CTCs that express EpCAM (epithelial cell adhesion molecule). This approach represents an advancement in CTC detection, enabling more accurate and reliable cancer diagnosis.

## 2. Experimental Part

### 2.1. Reagents and Materials

The following reagents were purchased from Sigma-Aldrich (St. Louise, MO, USA) and used as received: Sylgard 184, ethanol, Dulbecco’s phosphate-buffered saline (DPBS) with calcium chloride and magnesium chloride, 11-mercaptoundecanoic acid (MUA), 12-mercaptododecanoic acid, N-hydroxysuccinimide (NHS) ester, Avidin, gold nanoparticles 5 nm (752568), bovine serum albumin (BSA), N-[3-(trimethoxysilyl)propyl]ethylenediamine (AEAPTMS), and Tween-20. Anti-EpCAM (Anti-Human CD326) was purchased from eBioscience, San Diego, CA, USA.

### 2.2. Fabrication of the LFA Device

The LFA device was fabricated using a previously described method [25]. The channel pattern of the LFA device was sketched using AutoCAD. LFA consists of a 1 in. × 3 in. glass slide and a poly(dimethylsiloxane) (PDMS) layer containing four microchannels with a pattern on the upper surface. On the basis of the silicon master, a PDMS substrate was fabricated using soft lithography. Thoroughly mixed liquid pre-polymer (base/curing agent = 10:1) was cast on the silicon master. A PDMS-loaded aluminum foil bowl was put in a vacuum chamber to remove bubbles from PDMS. The aluminum foil bowl was cured at 65 °C for at least 4 h. PDMS was polymerized, forming a transparent elastic substrate after curing in the oven. The PDMS was then peeled off from the silicon master and trimmed to fit a microscope slide; holes were punched at the inlet and outlet. UV ozone bonded the glass microscope slide and PDMS substrate for 5 min. The image of the filters is shown in Figure S1A (in Supplementary Materials), and the device is shown in Figure S1B.

### 2.3. Functionalization of the LFA Device

To prepare the device for CTC capture, the LFA underwent a series of chemical modifications. Initially, the LFA was treated with a 1 wt.% solution of AEAPTMS in ethanol for 1 h at room temperature and subsequently washed with ethanol. Then, a 0.01 wt.% ethanol solution of NHS-functionalized gold nanoparticles (AuNPs) was applied to the device and incubated for 30 min at room temperature. The device was rinsed with ethanol to facilitate amide formation between NHS on AuNP and amine on AEAPTMS-coated PDMS channels. Finally, the channels were filled with a 20 µg/mL avidin solution in DPBS and left to incubate for 30 min, completing the preparation of the device for CTC capture.

The different shapes of nanoparticles have different surface properties and geometries, which can influence their binding affinity and targeting capabilities. The shape of nanoparticles can affect the hydrodynamic properties of the fluid within microfluidic devices. The shape of the nanoparticles can influence the flow behavior, shear stress, and velocity distribution of the fluid. By choosing the sphere shape, nanoparticles can be engineered to have enhanced interactions with CTCs, increasing their binding efficiency and specificity. This allows for better capture and detection of CTCs in microfluidic devices.

Then, 200 µL of DPBS was introduced to wash away excess avidin in the LFA device. The LFA device was functionalized with anti-EpCAM. A biotinylated anti-EpCAM solution of 70 µL with a concentration of 10 µg/mL was introduced to the LFA device, which was then incubated for 30 min. Before each experiment, the LFA device was passivated with a DPBS buffer containing 1% BSA (bovine serum albumin), followed by rinsing with DPBS to prevent nonspecific capture of normal blood cells.

### 2.4. Cell Culture

The L3.6pl pancreatic cancer cell line was obtained from Dr. Jose Trevino’s lab (University of Florida) [8], while the BxPC-3 pancreatic cancer cell line was acquired from the American Type Culture Collection (ATCC, Manassas, VA, USA). Following the recommended protocols, the cancer cell lines were expanded when they reached 85% confluence. These cells were cultured in DMEM (ATCC) supplemented with 10% fetal bovine serum (FBS; GIBCO of Thermo Fisher, Waltham, MA, USA) and 100 units/mL penicillin–streptomycin (Cellgro, Manassas, VA, USA) under incubation conditions of 37 °C with 5% CO_2_. The CCRF-CEM cells purchased from the ATCC were cultured in RPMI-1640 medium (ATCC) with 10% FBS and 100 units/mL penicillin–streptomycin. Before preparing the cell samples, the culture medium was removed from the flask, and DPBS was added to rinse the flask and remove any impurities. Trypsin EDTA (GIBCO, Fisher Scientific, Waltham, MA, USA), in a volume of 2 mL and concentration of 0.25%, was introduced, before incubating for 10 min to detach the cells from the flask. The growth medium of 6 mL was added to neutralize the cells. The detached cells were then rinsed with DPBS twice to remove impurities. Finally, the cells were resuspended in 1 mL of DPBS. CCRF-CEM cells were floating cells for cell sample preparation. The cells were withdrawn from the flask, rinsed with DPBS twice, and resuspended in 1 mL of DPBS. Following the manufacturer’s instructions, these cells were stained with Vybrant dyes (Thermo Fisher Scientific, Waltham, MA, USA). The dyed cells were then rinsed with DPBS before spiking into either DPBS buffer or blood samples. The infusion flow rate of the sample into the device varied from 0.5 mL/h to 2.0 µL/s. After the sample infusion, the device was washed by infusing 250 µL of DPBS.

### 2.5. Imaging Fluorescence

Fluorescence signals of tumor cells captured in the device were collected using an Olympus IX71 fluorescence microscope (Olympus America, Center Valley, PA, USA) equipped with a scientific-grade CCD camera (Hamamatsu C4742-80-12AG). For devices functionalized with antibodies, the captured cells were first trypsinized with one channel volume of 0.25% trypsin-EDTA for 10 min, followed by pumping DPBS from the outlet at 2.0 µL/s. The viability of released L3.6pl cells was determined by staining with 4% Trypan blue (Fisher Scientific, NH) following the manufacturer’s instructions.

### 2.6. Blood Processing

Clinical blood samples were collected from patients with metastatic pancreatic cancers at the UF Health Cancer Center. According to the protocol approved by the UF institutional review board (IRB), all specimens were processed within 3 h after the blood draw. Before the clinical test, two LFA devices were functionalized with anti-EpCAM and passivated with 1% BSA solution, as discussed above. The clinical samples were diluted twice (blood-to-DPBS ratio = 1:1) and infused into the antibody-functionalized device at 1.5 µL/s. A volume of 8 mL of diluted clinical samples were injected into two LFA devices in parallel (i.e., 4 mL for each device). After the infusion process, each device was washed with DPBS for impurity removal. Next, 50 µL of a 4% paraformaldehyde (PFA) solution was added to the device, which was incubated for 10 min for cell fixation. The device was washed with DPBS before adding 50 µL of a 0.2% Triton X-100 solution for 10 min to permeabilize the cell membrane. After washing, a mixture of 10 µg/mL anti-CD45–PE, 10 µg/mL anti-cytokeratin–FITC, and 500 nM DAPI was introduced to the device for staining, before incubating for 30 min. The device was then rewashed and mounted onto the fluorescence microscope stage for CTC enumeration. DAPI^+^, CD45^−^, and CK^+^ cells were counted as CTCs. Other cells, such as white blood cells (DAP^+^, CD45^+^, CK^−^, Sigma-Aldrich, St. Louis, MO, USA), red blood cells (DAPI^−^), and others (e.g., triple positive), were excluded.

## 3. Results and Discussion

**Figure 1:**
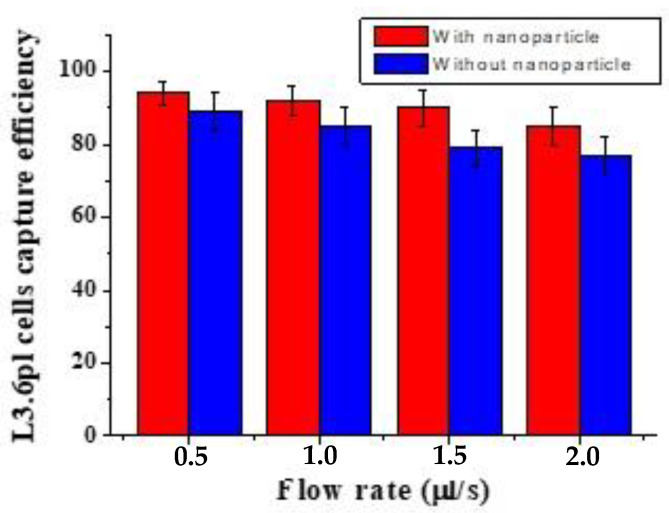
Cells captured in the LFA device under two conditions: with and without gold nanoparticle coated. L3.6pl cells were captured in the LFA device under different flow rates. Error bars show the range (n = 3).

To detect CTCs, we developed a microfluidic laminar flow device (LFA) and evaluated its capture performance with and without nanoparticles. We optimized the flow rate to determine the ideal conditions for capturing cells. We infused approximately 1000 fluorescence-labeled L3.6pl cells into the LFA to test the device’s efficacy. Figure 1 shows the cell capture ratio under different flow rates. The capture efficiency was optimized by varying the flow rate and comparing the capture performance in the presence and absence of nanoparticles. The results showed that the capture efficiency could reach over 94% ± 3.8% at a flow rate of 0.5 µL/s when nanoparticles were present. However, as the flow rate was increased from 0.5 to 1.5 µL/s, the capture efficiency decreased from 94% to 90%. The difference was not statistically significant at a low flow rate. Furthermore, Figure 1 shows that using AuNP-conjugated antibodies enhanced the capture efficiency of target cells compared to without nanoparticles. The capture rate of CTCs in the microfluidic system was calculated using the formula Cf/Cs × 100%, where Cf is the number of cancer cells counted under a fluorescence microscope, and Cs is the total number of cancer cells spiked into the DPBS buffer. These results suggest that the LFA device functionalized with nanoparticles could achieve highly efficient and specific capture of CTCs.

**Figure 2:**
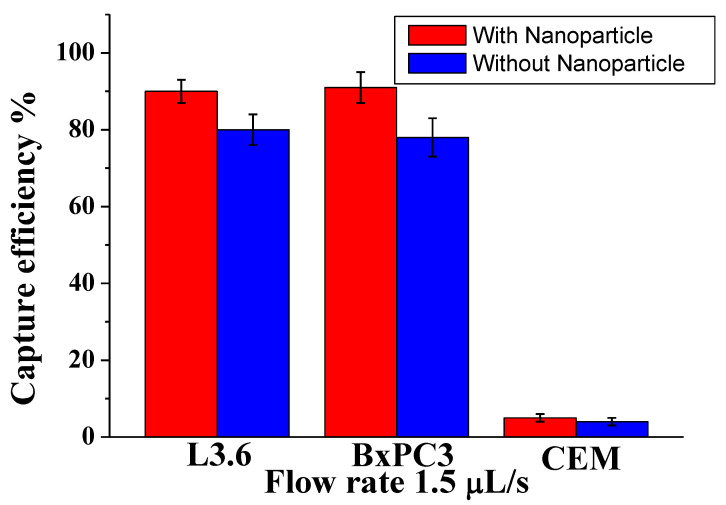
Comparison of capture efficiency using specific and nonspecific cells at an optimal flow rate of 1.5 µL/s. Error bars show the range (n = 3).

To demonstrate the efficiency among different types of cells with different sizes, we studied a mixture of pancreatic cancer cell lines: target L3.6pl cells, BxPC3 (EpCAM^+^), and control CCRF-CEM cells (EpCAM^−^). As shown in Figure 2, for L3.6pl cells (diameter 16 ± 3 µm), the capture efficiency was 91% ± 4.5% at 1.5 µL/s; for BxPC3 cells (diameter 16.0 ± 2 µm), the corresponding capture efficiency was around 88 ± 3.5%, while most control CEM cells (diameter 13.0 ± 2 µm) were removed by washing (capture of only 6.0% ± 2%). The possible reason is that the CCRF-CEM cells did not express EpCAM, and their sizes were slightly smaller than other cells tested. These results demonstrate that our device, conjugated with nanoparticles and anti-EpCAM, could efficiently isolate CTCs at low numbers by decreasing surface contact and increasing the binding efficiency. Furthermore, our methodology increased the surface roughness compared with the plain surface, allowing local topographic interactions between the aptamer and nanoparticle.

**Figure 3:**
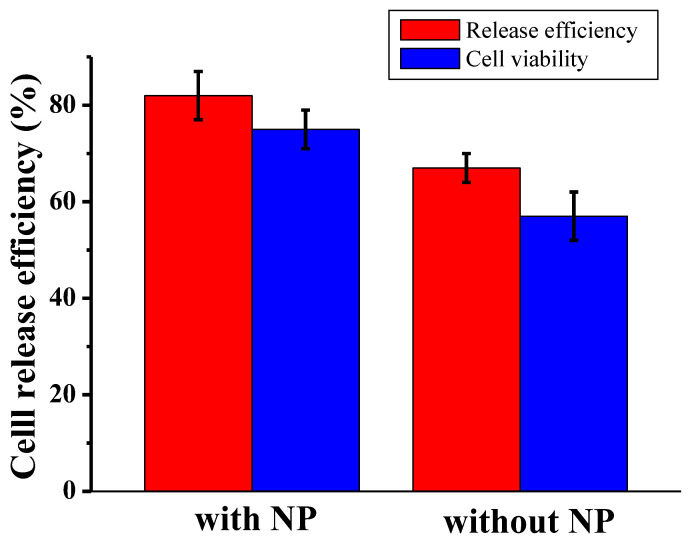
Release efficiency and cell viability of recovered L3.6pl cell in DPBS according to the presence and absence of nanoparticles, at a flow rate of 1.5 μL/s.

**Figure 4:**
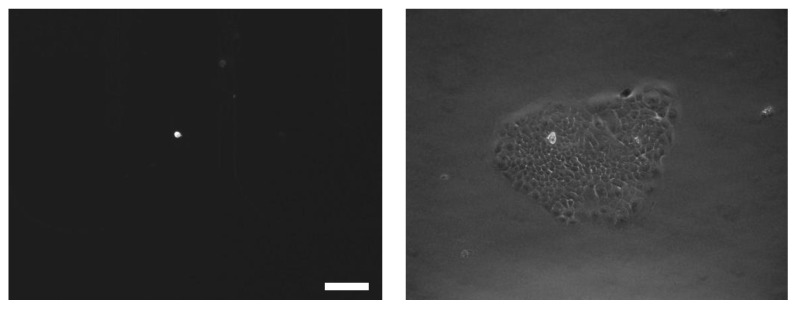
Bright-field images of released cells. Images of cells 2 days (**left**) and 7 days (**right**) post release (scale bar: 10 μm).

The release of captured cells, in both the presence and the absence of nanoparticles, was achieved through trypsinization. The efficiency of release was measured as the ratio of the number of cells released to the number of cells captured. Figure 3 shows the release efficiency achieved by flowing trypsin (1 mg/mL) for 30 min and the viability of the recovered CTCs. In the presence of nanoparticles, 80% ± 7% of the captured cells remained viable after the capture and release process, making them suitable for subsequent cellular analysis. In contrast, the device without nanoparticles showed a lower release efficiency and cell viability, with a decrease of approximately 20%. Here, we observed a strong correlation between the incorporation of the nanoparticle into the microfluidic chip and the observed improvements. Our assumption is that the shape of nanomaterials plays a crucial role in enhancing the screening efficiency of CTCs. In other words, by strategically designing flat microfluidic devices, we can optimize the screening performance. Our findings align with recent studies that demonstrated the profound impact of nanomaterial shapes on their biological performance, particularly in terms of cellular binding kinetics [26,27]. As expected, our results showed higher cell viability with the nanoparticles, oscillating between 87% and 93% with control and released cells. The released L3.6pl cells were cultured in a complete growth medium after capture and release. Figure 4 shows the cell culture at different timepoints after release.

**Figure 5:**
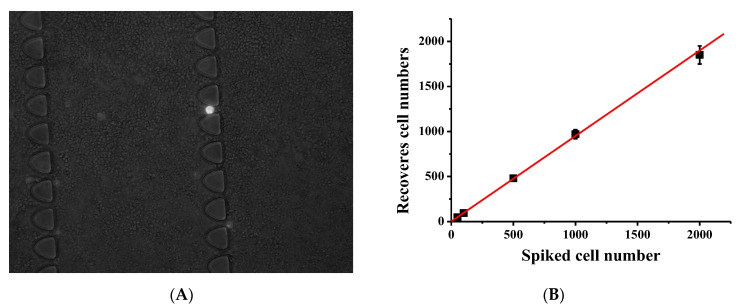
The capture of target cells from diluted blood. Different L3.6pl cells were spiked in 2 mL of twice-diluted whole blood and infused into the functionalized LFA device. (**A**) Comparison of spiked L3.6pl cells (white) and non-captured specific captured white blood cells (gray). (**B**) Calibration plot of cancer cell capture from whole blood and lysed blood with different cell concentrations at 1.5 μL/s; solid lines represent linear fitting. Error bars represent standard deviations (n = 3).

To assess the effectiveness of our CTC capture system in whole human blood, we conducted experiments using blood samples spiked with varying concentrations of L3.6pl cells under optimal conditions. Specifically, 2 mL of whole blood was spiked with L3.6pl cells at concentrations of 50, 100, 500, 1000, and 2000 cells per mL, and the samples were processed using the microfluidic device with a flow rate of 1.5 µL/s. Our results, presented in Figure 5B, demonstrate a strong linear correlation (R^2^ = 0.998, n = 3) between the number of spiked cells and the number of cells captured, with an average capture efficiency of 90% ± 3.5%. These findings suggest that our system can effectively capture CTCs from whole blood, even at low concentrations, and has the potential for use in clinical applications.

**Figure 6:**
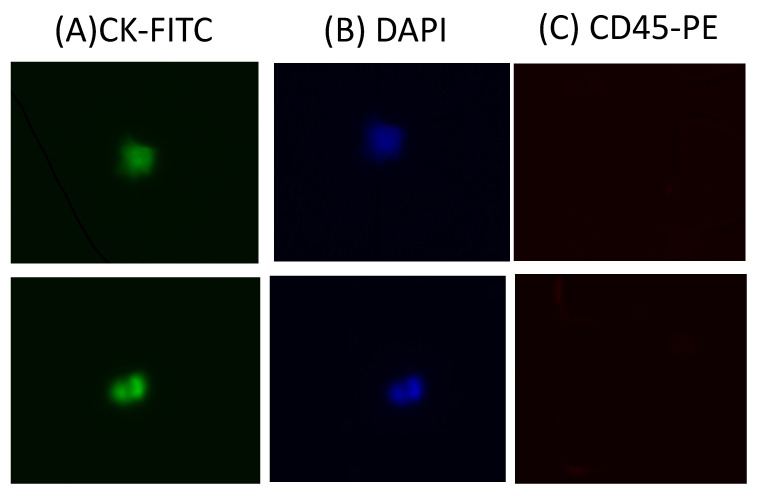
Sample images of captured CTCs: (**A**) CK–FITC channel image; (**B**) DAPI channel image; (**C**) CD45–PE channel image.

We evaluated the clinical utility of our methodology using blood samples obtained from a small group of patients with metastatic pancreatic cancer. Half of a blood sample (4 mL) was diluted with an equal volume of DPBS and then processed in the antibody-functionalized LFA device to reduce the effect of viscosity. This dilution also helped reduce blood viscosity. The cell sample was then introduced to the LFA device at a flow rate of 1.5 µL/s. After a DPBS washing step, captured cells were fixed with 4% paraformaldehyde (PFA) for 10 min and then permeabilized with 0.2% Triton X-100 for 10 min, followed by permeabilizing with 0.2% Triton X-100 for 10 min. Then, a mixture of labeling antibodies containing 10% of 10 µg/mL FITC anti-cytokeratin, 10% of 10 µg/mL PE anti-CD45, and 80% of 500 nM DAPI was introduced into the LFA device and incubated for 30 min. After DPBS washing, CTCs were enumerated under a microscope. To eliminate false-positive signals, we only considered cytokeratin-positive, CD45-negative, DAPI-positive (CK^+^/CD45^−^/DAPI^+^) cells as CTCs (Figure 6). Any other labeling formats were considered false-positive signals or cell debris. The average range of CTCs detected in our device was 3–8 CTCs/mL. Our results are in agreement with previous studies and demonstrate an improvement in the recovery efficiency of CTCs. [11,22,25], while also providing valuable insights into the design of LFA with modified nanoparticles, aimed at enhancing their capability to capture circulating tumor cells.

## 4. Conclusions

In this study, we presented a highly efficient method for capturing CTCs from patient blood samples using an LFA device modified with nanoparticles. By incorporating nanoparticles into the capture process, we significantly increased the capture efficiency of CTCs compared with devices without nanoparticles. Our experimental results demonstrated a capture efficiency of greater than 90%, with over 80% of captured cells successfully released while maintaining high viability levels. Moreover, our methodology allowed for processing 4 mL of blood within 20 min, making it a suitable technique for clinical applications. Our results suggest that nanoparticle conjugation and LFA can be promising strategies for capturing CTCs, which could be further tested and validated as part of clinical trial. The use of nanoparticles in microfluidic devices provides a shorter analytical time for CTC capture and detection, enhanced capture efficiency and specificity, and enhanced capability for sequential analysis after capturing and releasing resulting in a potential point of care for diagnosis and/or treatment monitoring for cancer patients.

## Data Availability

The data that support the findings of this study are available from the corresponding authors upon reasonable request.

## Supplementary Materials

The following are available online at https://www.mdpi.com/article/10.3390/bios13070706/s1, Figure S1: (A) SEM image of the device. The image shows the main coil channel and the arrangement of side filters. (B) Finished device.
