# Supplementary material for: Gold Nanoparticle-Based Microfluidic Chips for Capture and Detection of Circulating Tumor Cells
Source: Biosensors (Basel). 2023 Jul 4;13(7):706. doi: 10.3390/bios13070706 (PMC10377447; doi:10.3390/bios13070706)
Supplement: Supplementary file 1 [file biosensors-13-00706-s001.zip › biosensors-2392264-supplementary.pdf]

Supplementary Material

Gold Nanoparticle-Based Microfluidic Chips for Capture and Detection of Circulating Tumor Cells

Valber A. Pedrosa [1,*], Kangfu Chen [2], Thomas J. George [3] and Z. Hugh Fan [2,4]

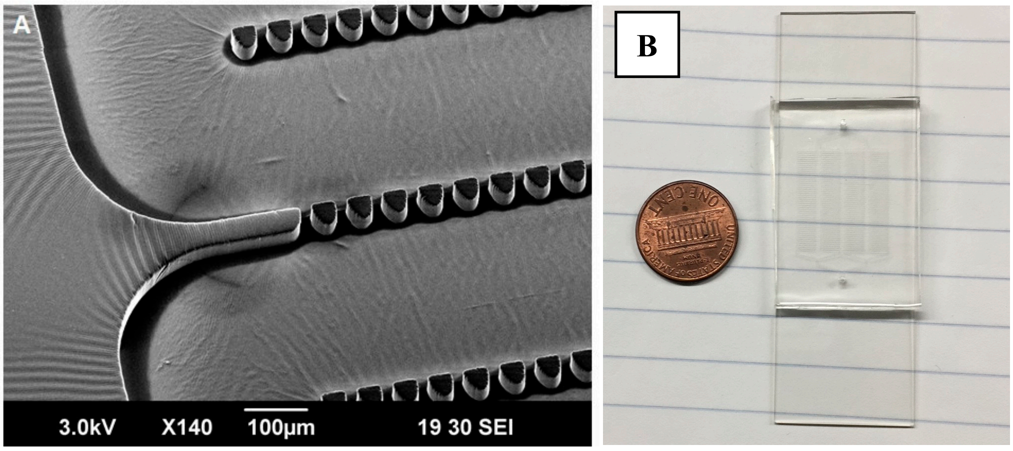

Figure S1. (**A**) SEM image of the device. The image shows the main coil channel and the arrangement of side filters. (**B**) Finished device.
